# Woody lianas increase in dominance and maintain compositional integrity across an Amazonian dam-induced fragmented landscape

**DOI:** 10.1371/journal.pone.0185527

**Published:** 2017-10-17

**Authors:** Isabel L. Jones, Carlos A. Peres, Maíra Benchimol, Lynsey Bunnefeld, Daisy H. Dent

**Affiliations:** 1 Biological and Environmental Sciences, University of Stirling, Stirling, United Kingdom; 2 School of Environmental Sciences, University of East Anglia, Norwich, United Kingdom; 3 Universidade Estadual de Santa Cruz, Ilhéus, Bahia, Brazil; 4 Smithsonian Tropical Research Institute, Apartado, Balboa, Panama; Chinese Academy of Forestry, CHINA

## Abstract

Tropical forest fragmentation creates insular biological communities that undergo species loss and changes in community composition over time, due to area- and edge-effects. Woody lianas thrive in degraded and secondary forests, due to their competitive advantage over trees in these habitats. Lianas compete both directly and indirectly with trees, increasing tree mortality and turnover. Despite our growing understanding of liana-tree dynamics, we lack detailed knowledge of the assemblage-level responses of lianas themselves to fragmentation, particularly in evergreen tropical forests. We examine the responses of both sapling and mature liana communities to landscape-scale forest insularization induced by a mega hydroelectric dam in the Brazilian Amazon. Detailed field inventories were conducted on islands created during reservoir filling, and in nearby mainland continuous forest. We assess the relative importance of variables associated with habitat fragmentation such as area, isolation, surrounding forest cover, fire and wind disturbance, on liana community attributes including abundance, basal area, diversity, and composition. We also explore patterns of liana dominance relative to tree saplings and adults ≥10 cm diameter at breast height. We find that 1) liana community composition remains remarkably similar across mainland continuous forest and islands, regardless of extreme area- and edge- effects and the loss of vertebrate dispersers in the latter; and 2) lianas are increasing in dominance relative to trees in the sapling layer in the most degraded islands, with both the amount of forest cover surrounding islands and fire disturbance history predicting liana dominance. Our data suggest that liana communities persist intact in isolated forests, regardless of extreme area- and edge-effects; while in contrast, tree communities simultaneously show evidence of increased turnover and supressed recruitment. These processes may lead to lianas becoming a dominant component of this dam-induced fragmented landscape in the future, due to their competitive advantage over trees in degraded forest habitats. Additional loss of tree biomass and diversity brought about through competition with lianas, and the concurrent loss of carbon storage, should be accounted for in impact assessments of future dam development.

## Introduction

Fragmentation of primary tropical forests results in the loss of integrity of biological communities isolated in remnant forest fragments [1–3]. Synergistic area- and edge-effects can lead to biodiversity loss, changes in community composition, and declines in ecosystem functioning of forest fragments [1]. Tropical forests are a global sink of atmospheric carbon [4]. Yet once fragmented, remnant tree communities experience rapid turnover, significantly reducing the carbon storage potential of forest fragments [5]. Lianas (woody vines) are well-adapted to the harsh environmental conditions associated with fragmented landscapes, and compete with trees at all life stages [6]. Thus, through competition for resources with trees, lianas may intensify the loss of tree diversity and reduce carbon storage across fragmented landscapes [7,8]. Few studies, however, have assessed the assemblage-level effects of forest fragmentation on lianas. Improving our understanding of liana assemblage responses to insularization is therefore essential to assess the long-term impacts of tropical forest fragmentation on forest composition and carbon storage [9].

Lianas are ubiquitous in tropical forests, contributing to species diversity, ecosystem functioning and dynamics, forest architecture and arboreal connectivity [10,11]. Lianas can comprise up to 44% of woody species [12] and 10–45% of woody stems present within tropical forest communities [13]. Lianas compete with trees for light, water and nutrients [6], reducing tree reproduction, recruitment and diversity [14,15], growth and survival [16,17]. Studies from Panama have shown that the competitive strength of liana-tree interactions may be greater than that between trees [18]. Although all tree species may be affected by competition with lianas [19], the effects are more detrimental to slow-growing shade-tolerant trees than fast-growing pioneer species, due to higher rates of crown infestation and physical damage [14,16]. Lianas have a competitive advantage over trees in high light and low moisture conditions [20], making them a common feature of degraded and secondary tropical forest habitats [21]. Lianas have been found to arrest successional processes in forest gaps by stalling tree recruitment [22], highlighting the profound effect lianas may have in determining future tree assemblages as tree communities respond to fragmentation through time.

Unlike trees, lianas do not channel resources into trunk diametric growth to reach forest canopies. Instead, they use trees as trellises and invest resources into rapid vertical growth and leaf production [10,23]. Liana biomass is therefore lower than that of trees, and represents an estimated 4% to 14% of total forest above-ground biomass [24,25]. Across the Neotropics, studies have shown that the abundance and biomass of lianas is increasing [9,20,26,27]. If slow-growing high-carbon-storing tree species are particularly impacted by liana proliferation, this may lead to a reduction in tropical forest carbon storage [7,23,28]. Furthermore, because lianas allocate more carbon to leaf production than trunk growth, increasing liana abundance and biomass could shift the carbon balance in tropical forests from long-term carbon sequestration in woody biomass, towards more rapid turnover in leaves [7]. An increase in litterfall from lianas may also accelerate below-ground carbon cycling through priming effects, further reducing total carbon storage within tropical forests [29].

Primary tropical forests are becoming increasingly fragmented and degraded due to deforestation and land-use change [30]. The flooding of tropical forests for hydropower is an emerging driver of tropical forest fragmentation and degradation [31,32]. Tropical dams are controversial in terms of the area of land flooded and significant greenhouse gas emissions from reservoirs [33], pervasive loss of species from reservoir islands [34], and inadequate carbon cost/benefit analyses [32]. During reservoir filling, tropical forest habitat becomes isolated on land-bridge islands within an open-water matrix, leading to the most extreme scenario in terms of fragmentation effects [3,35–38]. All reservoir land-bridge islands, regardless of their area, exhibit long-term species loss, but rates are higher on small islands which pay their extinction debts faster [34]. Tropical forest tree communities isolated on reservoir land-bridge islands undergo rapid compositional change, with functional shifts, declines in abundance of many species, and local extinctions [3,35,39,40]. Loss of biotic seed dispersal due to diminished vertebrate communities may further alter future floristic composition and carbon storage potential of insular tree assemblages [41,42].

In this study, we explore community-wide liana responses to landscape-scale fragmentation induced by the Balbina mega-dam in Central Brazilian Amazonia. We consider both sapling and mature lianas, and investigate a number of metrics including liana abundance, basal area, dominance relative to trees, and seed dispersal mode, as well as diversity and community composition. We relate these community attributes to environmental variables widely associated with fragmented systems at plot-, site-, and landscape-scales including island area, isolation, surrounding forest cover, and degree of fire and wind disturbance.

Given the propensity for lianas to establish in degraded habitats, we hypothesise that 1) both sapling and mature lianas will increase in abundance, basal area, and dominance relative to trees in the most disturbed island habitats, where synergistic area- and edge-effects are most pronounced. This is in contrast to tree communities on islands, which have rapidly eroded across the Balbina archipelago [3]. When considering the sapling layer we also expect that 2) the abundance of vertebrate-dispersed lianas will be lower in disturbed habitats due to the loss of biotic seed dispersal capacity caused by widespread local extinctions of animal seed dispersers [38,41–43]. As edge-and area-effects become less pronounced, such as when island area is greater and fire disturbance lower, forest communities appear to more closely resemble those found in mainland continuous forest [3,38]. Thus, we expect that 3) the diversity and community composition of lianas will converge with those of mainland continuous forest as area- and edge-effects diminish.

## Methods

### Study site

The Balbina Hydroelectric Dam in Central Amazonia (1°010–1°550 S; 60°290–59°280 W) was closed in 1986, flooding ~3129 km^2^ of lowland primary wet tropical forest, creating 3546 forest islands within the reservoir, ranging in size between 0.2 and 4878 ha [3,38]. These forest islands have never been logged, and both the islands and the mainland extending east of the former Uatumã River bank are strictly protected as part of the Reserva Biológica do Uatumã, the largest biological reserve in Brazil. Such measures have largely prevented subsequent anthropogenic disturbance. However, in 1997 a fire was accidentally started in the unprotected portion of the Balbina reservoir, which spread between some islands; mainland continuous forest areas were unaffected.

**Fig 1:**
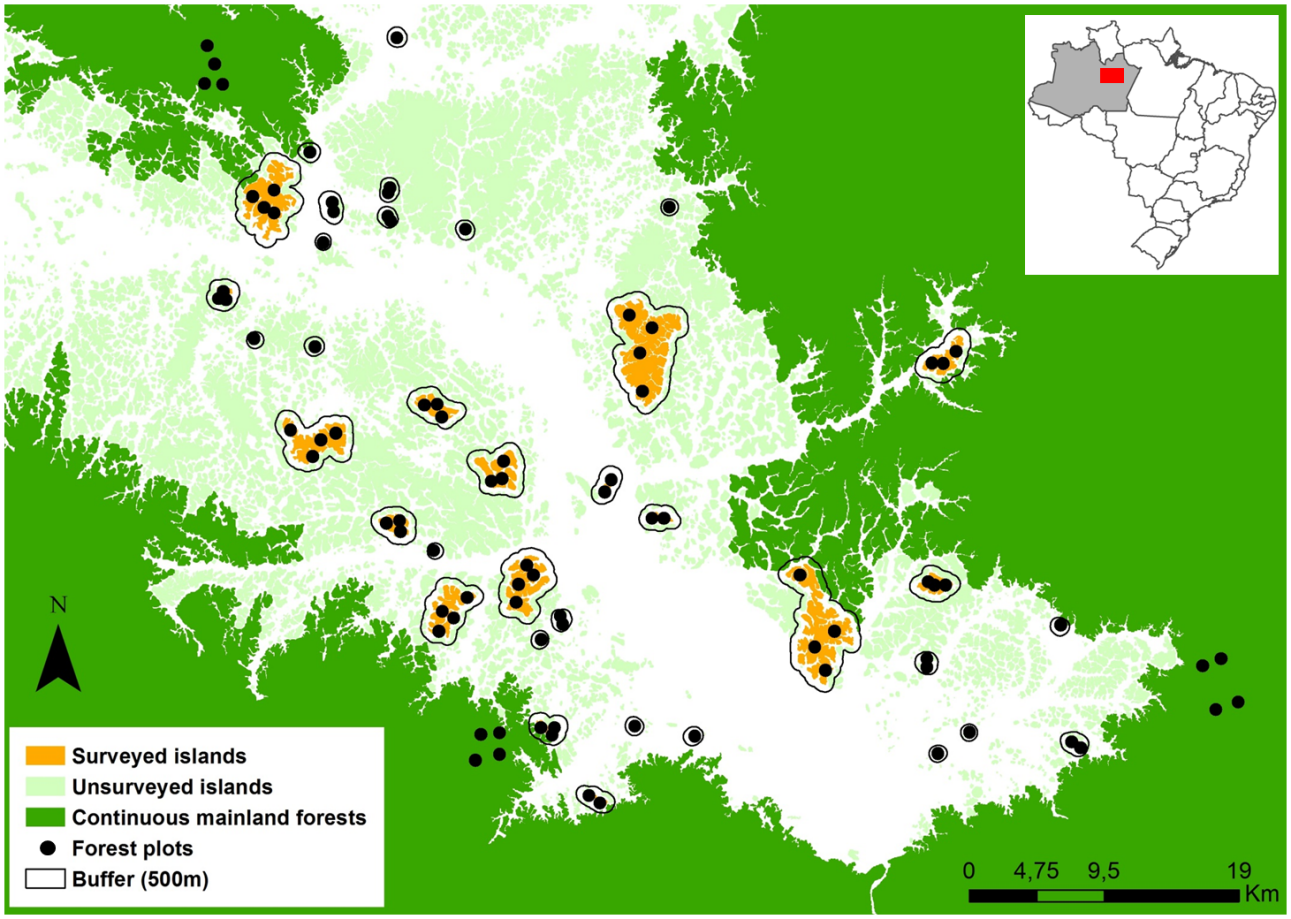
Location of permanent census plots within the Balbina Hydroelectric Dam (Amazonas, Brazil). The 89 permanent plots are nested within 36 spatially-independent islands and in three continuous mainland forest sites. The percentage of forest cover (‘Cover’) within each 500 m buffer was used as one of seven ecologically-relevant variables used within analyses of liana assemblages.

We selected 36 spatially-independent focal islands, and three widely-spaced mainland continuous forest sites adjacent to the archipelago (Fig 1) using two cloudless georeferenced Landsat ETM+ scenes from 2009 (230/061 and 231/061). Islands were selected on the basis of their size (0.83–1690 ha; mean ± SD = 210.7 ± 392.1), isolation, i.e., distance to the nearest mainland (0.04–17.73 km; mean ± SD = 4.9 ± 4.4), spatial distribution (keeping a minimum distance of 1 km from one another), and to span the gradient of fire severity. In 2011, one to four 0.25 ha (10 m x 250 m) rectangular permanent plots were established on 34 forest islands and in all three mainland continuous forest sites, creating a preliminary network of 87 forest plots. In 2014, one additional 0.25 ha plot was established on each of two small islands (< 10 ha) bringing the total number of permanent plots to 89: 77 plots are nested within 36 islands, and 12 plots are nested within three mainland continuous forest sites surrounding the reservoir (Fig 1).

### Liana and tree inventories

We performed surveys to record both saplings and mature woody lianas, and sapling and adult trees ≥10 cm diameter at breast height (DBH, ~130 cm). Liana and tree saplings were surveyed in 89 forest plots in 2014, whereas adults of both groups were sapling in 87 plots in 2012. Woody liana saplings ≤2 cm diameter and ≥1 m height were recorded within 0.025 ha (1 m × 250 m) subplots, which followed the central axis of each of the 89 permanent plots. In 11 of the 89 plots, a reduced area was sampled for saplings (0.015–0.02 ha) compared to the standard 0.025 ha. No saplings had reached the forest canopy. Saplings were identified to genus level by A.E.S. Santos, an expert botanist with >20 years of herbarium and field experience working in Central Amazonia, and extensive experience of the Balbina woody flora [3]. We broadly classified liana genera by dispersal mode (either biotically- or abiotically-dispersed) by assessing morphological characteristics of fruits and seeds associated with dispersal, from the literature and personal observation of A.E.S Santos and the co-authors. For example brightly coloured fleshy fruits tend to be biotically-dispersed, whereas dry capsules or winged seeds tend to be abiotically-dispersed [44,45]. Tree sapling abundances were also obtained for each of the 89 0.025 ha sapling subplots, using the same survey method employed for liana saplings. Sapling surveys were carried out under permit No. 45849 issued by the Instituto Chico Mendes de Conservação da Biodiversidade (ICMBio/MMA).

Lianas ≥2.5 cm DBH were recorded within 87 of the 89 0.25 ha plots, following the measurement protocols of [46]. As 80% of lianas ≥2.5cm diameter are likely to have reached the forest canopy, we refer to these as ‘mature lianas’ [47]. Mature lianas were not identified. During the same survey period, all live adult trees ≥10cm DBH were inventoried within the 87 quarter-hectare plots. Adult trees ≥10cm DBH were identified to species-level by A.E.S. Santos, and by identification of voucher specimens at the National Institute for Amazon Research (INPA). Full details of adult tree inventories are presented in [3].

### Liana community attributes

We investigated six ecological attributes related to liana communities at the plot-scale: 1) abundance, 2) basal area, 3) proportion of biotically- vs. abiotically-dispersed individuals, 4) dominance relative to trees, i.e. abundance of lianas: abundance of trees (e.g. [27]), 5) liana genera diversity and 6) community composition. Diameter measurements were used to calculate the basal area of mature lianas ($Ba\left(m^{2}\right)=pi\;\times \frac{DBH{\left(cm\right)}^{2}}{4000}$) which were summed to give plot-level estimates. Taxonomic information was only available for liana saplings, therefore analyses of seed dispersal mode, diversity and community composition were conducted only for this size class. To compare liana community diversity among plots, we calculated Fisher’s α diversity values, as Fisher’s α is a robust metric of assemblages with varying numbers of individuals [21].

### Environmental variables

We used and processed 28 tiles of Rapid-Eye high-resolution (5 m pixel) imagery covering an area of 698,000 ha of the Balbina landscape to quantify seven ecologically important environmental variables at the scale of whole landscapes, sites and individual plots that were identified as potentially important drivers of observed patterns of sapling and mature liana communities (see [3] for more details). Following a semi-supervised image classification using ArcMap [48], we obtained four land cover classes (closed-canopy forest, open-canopy forest, bare ground, and water) across the landscape. Thus, at the landscape-scale, we derived estimates of the percentage of forest cover (‘Cover’, %) within a 500 m buffer extending from the perimeter of each island and mainland sites, considering the sum of both closed-canopy and open-canopy forests. Cover provides a measure of landscape connectivity, encompassing both the degree of isolation from, and extent of, surrounding forested habitat.

Given that island area and isolation are central to the island biogeography paradigm [49,50], we measured both variables at the site-scale. Using ArcGIS we used the ‘calculate geometry tools’ and ‘spatial analyst tools’ to calculate the area of each island (‘Area’, in hectares), and the shortest linear distance from the perimeter of a focal island to continuous mainland forest (‘Isolation’, metres) respectively. The extent to which fires have penetrated the forest understorey of each site (‘Fire’) was defined as an ordinal factor with scores of 0–3. No mainland continuous forest sites had been burned (score 0), but all islands had been burned to different extents (scores 1–3).

Finally, at the plot-scale, we obtained the distance to the nearest edge (‘D_EDGE_’, metres), which was calculated as the mean shortest linear distance between each census plot and the forest edge, and provides information on how close plots are to the forest-water boundary where the impacts from edge-effects are most severe. Balbina experiences powerful convective windstorms, which have a known prevailing direction. We therefore used the angular difference (0–90°) between the main axis of each rectangular plot and the median prevailing windstorm direction (‘Wind’), where higher Wind values indicate that the prevailing wind direction is increasingly perpendicular to plots. Windstorms that hit plots along their entire length are thought to cause more structural disturbance across a greater area of the plot, potentially driving patterns of liana abundance and diversity. As terrain steepness can influence rates of species turnover and the variety of edaphic niches present [24], we obtained a measure of plot-scale topographic heterogeneity by calculating the difference in maximum and minimum elevation using Shuttle Radar Topography Mission—SRTM—raster data (‘Slope’, metres).

### Data analysis

#### Liana community attributes

All analyses were conducted using ‘R’ (version 3.3.2 [51]). We initially tested for overall differences in liana community attributes (abundance, basal area, seed dispersal mode, dominance relative to trees, and diversity) between islands and mainland sites using one-way ANOVAs.

The effects of environmental drivers on liana community attributes across the Balbina archipelago were explored using both linear mixed models (LMMs) and generalised linear mixed models (GLMMs) to account for a nested sampling design (‘lmer’ and ‘glmer’ within the ‘lme4’ R package; [51]). Prior to analysis, all continuous environmental variables were rescaled (centred and divided by two standard deviations [52]), using the 'rescale' function within the 'arm' R package [53], to enable comparison of the relative effect sizes of each environmental variable on response variables.

Abundance data were modelled using a GLMM with Poisson error structure (link = “log”). Relative dominance and the proportion of biotically- vs. abiotically-dispersed stems were modelled using binomial GLMMs (link = “logit”), to enable the data to be modelled while accounting for sample size. Basal area and Fisher’s α values were log-transformed and modelled using a LMM with a Gaussian error structure. All regression analyses included only island plots, both because any potential mainland/island effect is confounded with Fire (all islands had been burned to some degree while none of the mainland sites had been burned), and to avoid assigning arbitrary values for Area and Isolation for mainland continuous forest sites, which may have artificially influenced model fits.

Before fitting each model, a pair-wise correlation matrix was inspected. If any pair of environmental variables showed high co-linearity (r > 0.7; Pearson’s correlation coefficient) then one variable from the pair was removed from the model. Additionally, variance inflation factors (VIFs) for each variable were inspected for each model, with variables retained if VIF < 4 [54]. D_EDGE_ was consistently highly correlated with Area, and failed to enhance the amount of variance explained, so was excluded from all linear regressions. All full models included Area, Isolation, Cover, Wind, Slope, and Fire as fixed effects. Slope was not included in models investigating seed dispersal mode, as it was deemed negligibly biologically meaningful in determining the proportion of biotically- vs. abiotically-dispersed stems within plots.

The 77 island plots were nested within 36 islands, thus, ‘island’ was fitted as a random effect to account for potential pseudo-replication of plots on the same island. ‘Sampling area’ was also fitted as a random effect to account for potential variation arising from the different areas sampled in a small number of plots. If there was no difference in effect between two levels of burn severity, the two levels in question were collapsed creating a binary Fire variable. If there was no difference in effect among all three levels of Fire, all islands were effectively equal in terms of the burn severity effect, and Fire was removed from the model. Full models were simplified through stepwise deletion of non-significant terms (t-value >2 or <-2) and inspection of AIC values [55], and the best model confirmed by inspecting both AIC and AIC weight after model selection performed in the ‘MuMin’ R package [56]. To ascertain the relative importance of each environmental variable on response variables, coefficient estimates for each environmental variable were extracted from full models, and 95% confidence intervals were calculated.

#### Community composition

To determine if island and mainland sites were spatially auto-correlated, a Mantel test was performed on two dissimilarity matrices of pair-wise distances between islands and mainland sites, using Euclidean geographic distance and Hellinger-transformed species composition [57,58].

The degree of community composition similarity between island and mainland plots was visually inspected using non-metric multidimensional scaling (NMDS) ordination [59]. We performed unconstrained NMDS ordinations of liana genera within plots using two indices: the abundance-based Morisita-Horn dissimilarity index, which is particularly suited to communities that may have been insufficiently sampled [60], and the incidence-based Jaccard dissimilarity index. We then performed further abundance- and incidence-based ordinations excluding mainland plots, to explore potential patterns in community dissimilarity among island plots. We fitted the rescaled environmental variables used in the regression analyses to ordinations of island plots, and retained variables that were significant (*p* < 0.05) in explaining plot positions within ordination space. All NMDS was performed using ‘metaMDS’ within the ‘vegan’ R package [58].

We used permutational multivariate analysis of variance (perMANOVA) to statistically test for differences in abundance- and incidence-based community composition across all island and mainland plots, while accounting for our nested sampling design. perMANOVA was also used to assess the relative importance of site- and landscape-scale environmental variables in driving differences in abundance- and incidence-based community composition among island plots. To obtain significance values for individual environmental variables, variables were added sequentially to different models, and the significance reported is the value generated when the corresponding variable was added last. perMANOVA was performed using ‘adonis’ within the ‘vegan’ R package [58].

## Results

### Liana inventories

A total of 2,688 liana saplings belonging to 31 genera were recorded across the 89 0.025-ha subplots (S1 Table). Of the 31 genera, 16 were biotically-dispersed (1,016 stems), and 15 abiotically-dispersed (1,850 stems; S2 Table). The number of stems per genus ranged from 1 to 553 (mean = 93); five genera were represented by singletons. Between 9 and 78 liana saplings representing 3 to19 genera were recorded in each subplot. Some 40% of liana saplings (*N* = 1,105) belonged to two abiotically-dispersed genera, *Macherium* (Fabaceae) and *Memora* (Bignoniaceae). A total of 2,261 mature lianas were recorded, with between 0 and 69 individuals recorded per 0.25 ha plot (S1 Table). Mature lianas were not present in two plots located on small (< 10 ha) islands. The majority of mature lianas (78%, *N* = 1,766) ranged between 2.5 and 10 cm DBH.

### Liana community attributes

**Fig 2:**
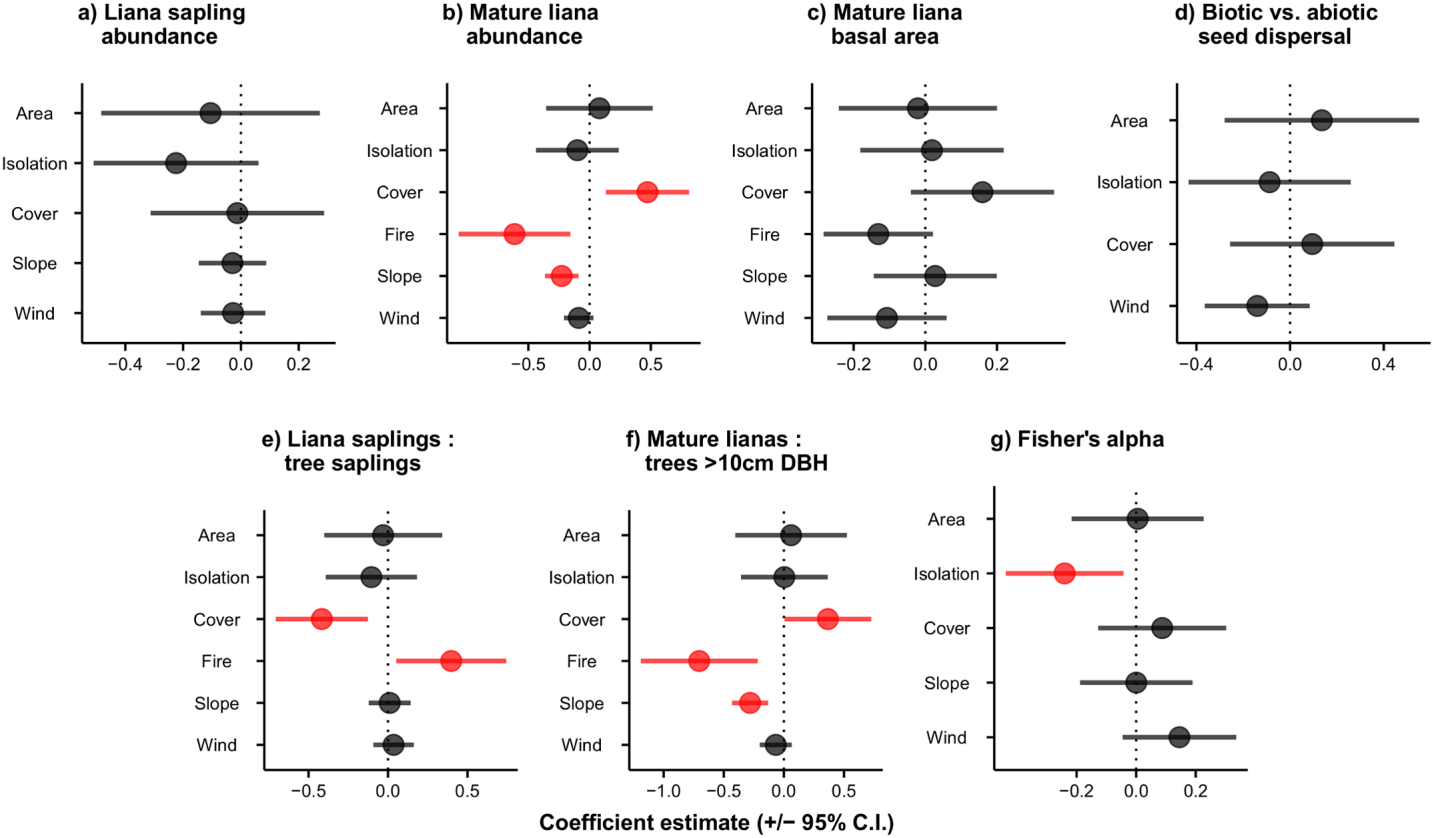
Standardised effect sizes of environmental variables associated with sapling and mature liana communities on forest islands. Coefficient estimates from maximal models are plotted with 95% confidence intervals. Points in red indicate that coefficient estimates and confidence intervals do not overlap zero.

**Fig 3:**
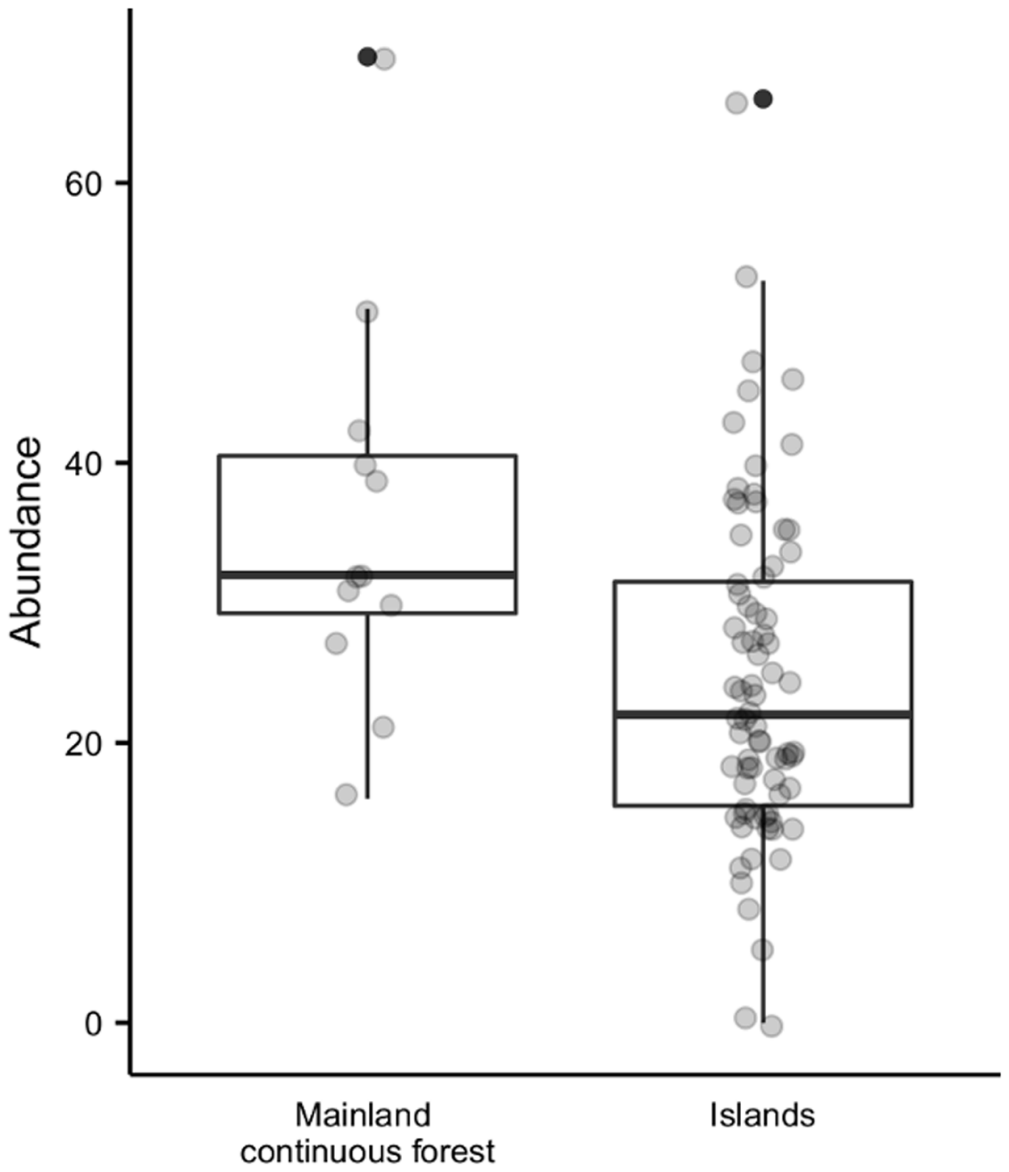
Abundance of mature lianas within island and mainland forest plots. Circles represent total numbers of mature lianas within each surveyed plot.

**Fig 4:**
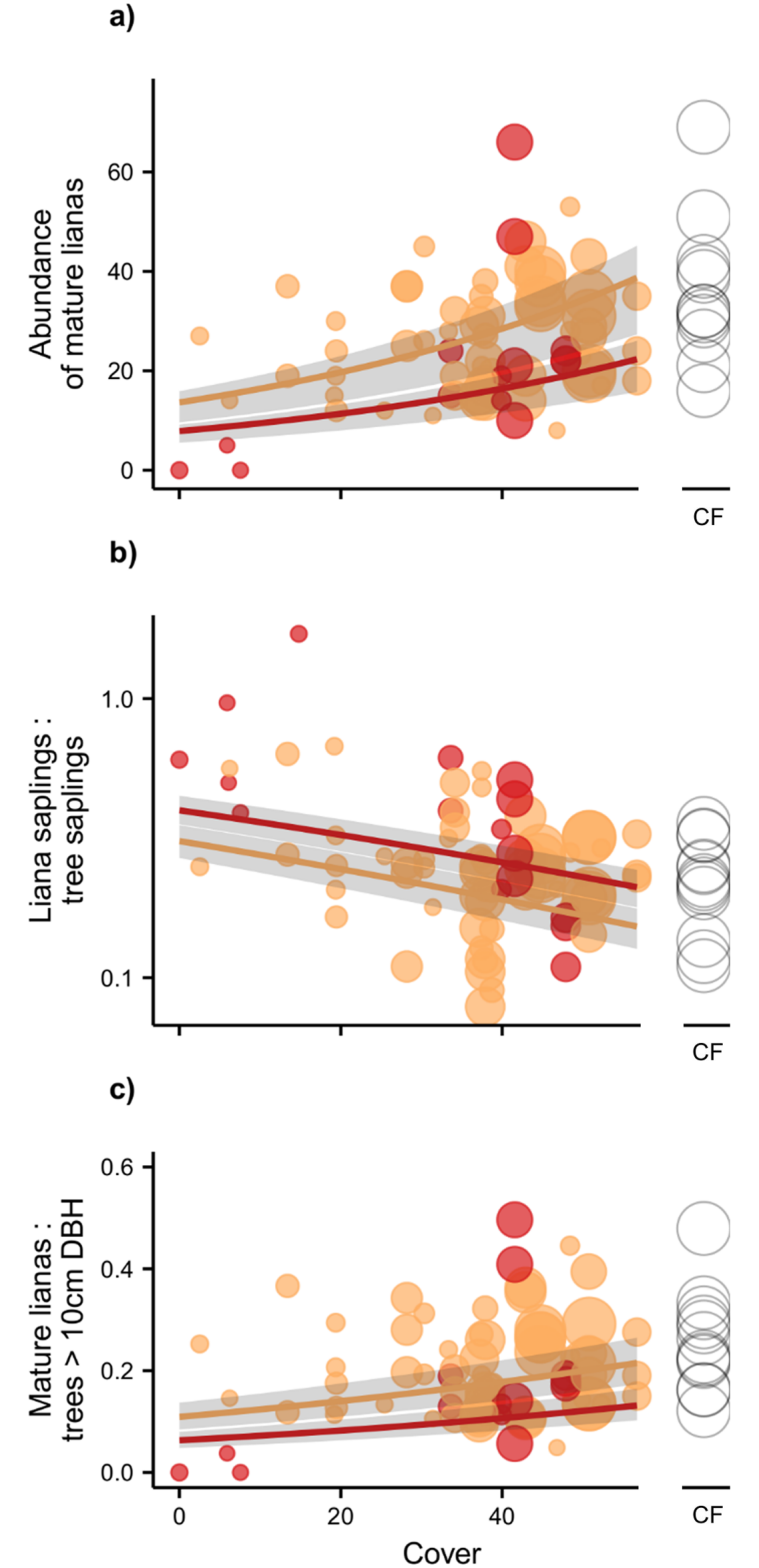
Abundance and relative dominance of lianas. The (a) abundance, (b) relative dominance of liana saplings to tree saplings, and (c) relative dominance of mature lianas to trees ≥10 cm DBH, as a function of the amount of neighbouring forest cover. Plot-level data are plotted as circles, scaled in size by island area. In each case a binary Fire variable was used, where low-moderate burn severity (scores 1 and 2; orange circles) were collapsed into a single level and compared to severe burning (score 3; red circles). Lines are predicted values, with grey shading indicating 95% confidence intervals. Data for mainland continuous forest plots (CF; open circles) were not included in model fits but are shown here for comparison.

There was no overall difference between liana sapling abundance in island vs. mainland plots (S3 Table), and among island plots, abundance was not significantly related to any environmental variables (Fig 2a). The abundance of mature lianas was, however, significantly lower on islands compared to the mainland (Fig 3; S3 Table). On islands, the abundance of mature lianas was significantly reduced in severely burned areas and when terrain was steeper, but increased with higher levels of Cover (Figs 2b and 4a; Table 1). The total basal area of mature lianas did not significantly differ between islands and the mainland (S3 Table), nor did it vary among island plots (Fig 2c). There was also no overall difference in the proportion of biotically- vs. abiotically-dispersed stems between islands and the mainland (S3 Table), or among islands (Fig 2d).

**Table 1:** Model selection table. Model selection table of the most parsimonious model for different attributes of woody liana communities across 77 island plots, based on LMMs or GLMMs. Coefficient estimates for significant fixed effects within ‘best models’, with ‘site’ and ‘sampling area’ as random effects, are presented; *t*-values >2 or <-2 were treated as significant. AICc values and AICc weights of final models are presented. Dashes indicate lack of any significant predictors.

Community characteristic	Fixed effects	Estimate	Standard error	*t*-value	AIC_c_	AIC_c_ weight
Abundance of liana saplings	-	-	-	-	-	-
Abundance of mature lianas	Intercept	3.16	0.08	32.45	651.3	0.43
	Cover	0.49	0.16	3.01		
	Slope	-0.23	0.07	-3.28		
	Fire	-0.65	0.24	-2.7		
Total basal area of mature lianas	-	-	-	-	-	-
Proportion of biotic vs. abiotically dispersed stems	-	-	-	-	-	-
Relative dominance of liana saplings	Intercept	-1.38	0.08	-16.97	583.0	0.64
	Cover	-0.44	0.13	-3.32		
	Fire	0.4	0.17	2.22		
Relative dominance of mature lianas	Intercept	-1.67	0.1	-16.34	641.7	0.59
	Cover	0.38	0.17	2.28		
	Slope	-0.28	0.08	-3.65		
	Fire	-0.6	0.22	-2.89		
Fisher’s α	Intercept	1.58	0.05	33.01	97.0	0.82
	Isolation	-0.23	0.09	-2.49		

There was no significant difference in the dominance of liana saplings relative to tree saplings in islands vs. mainland plots (S3 Table). However, liana saplings were significantly more dominant than tree saplings in severely burned island plots, and significantly less dominant where neighbouring forest cover was greater (Figs 2e and 4b; Table 1). The dominance of mature lianas relative to trees ≥10 cm DBH was significantly lower on islands compared to mainland plots (S3 Table). On islands, mature lianas became increasingly dominant relative to trees in more forested landscapes (Fig 4c; Table 1), and significantly less dominant in plots with steeper slopes that experienced a history of severe fires (Fig 2f; Table 1). Liana diversity (Fisher’s α) at the genus level was not significantly different between island and mainland plots (S3 Table), but liana diversity significantly declined in more isolated islands (Fig 2g; Table 1).

### Community composition

**Fig 5:**
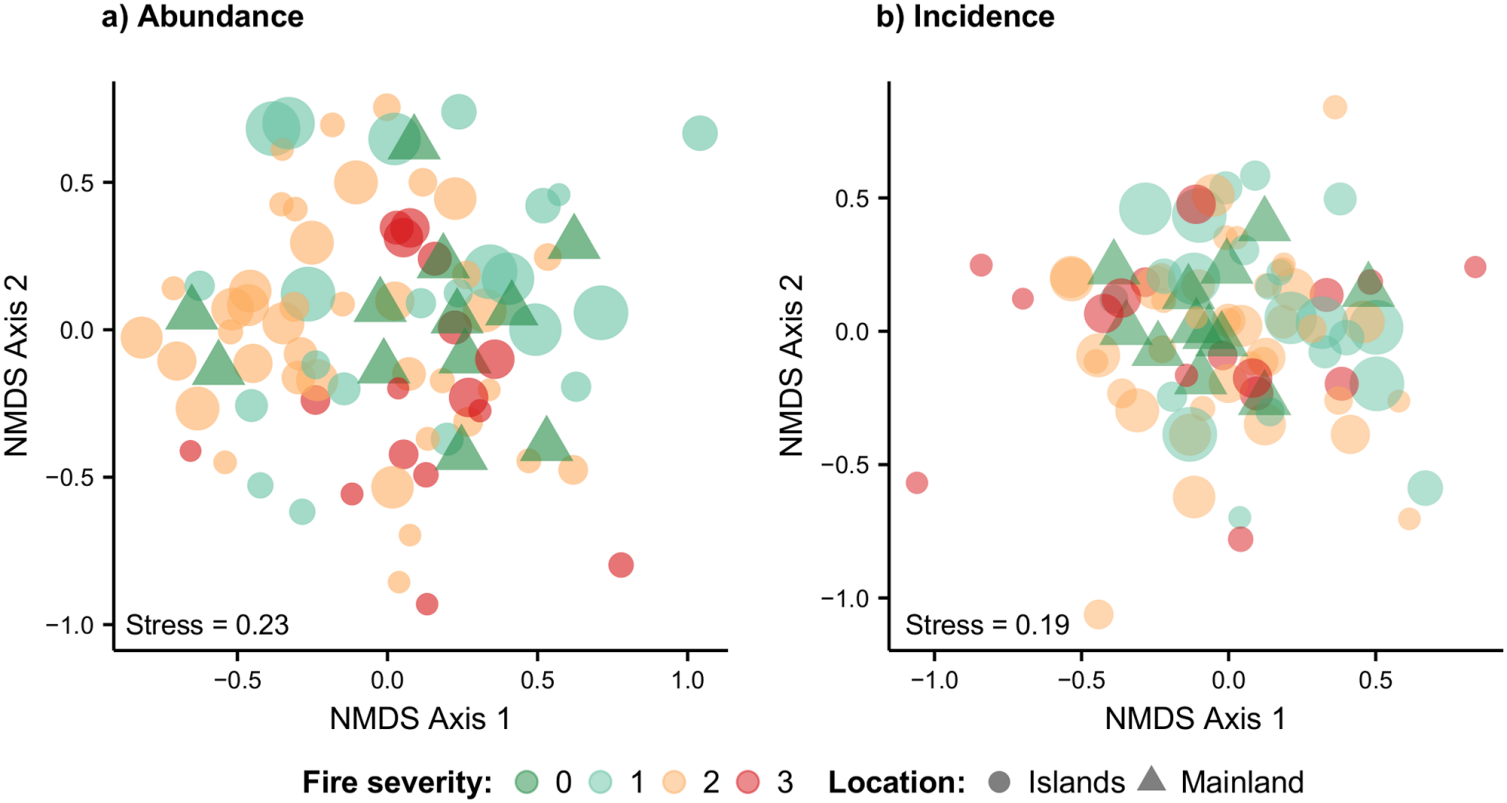
NMDS ordination of liana sapling communities. NMDS ordination of all island and mainland continuous forest plots using (a) the abundance-based Morisita-Horn dissimilarity index, and (b) the incidence-based Jaccard dissimilarity index. Plotted circles are scaled by island area. Points are colour-coded by burn severity: 0 = no burn, 1 = light burn, 2 = moderate burn, and 3 = severe burn. The proximity of plotted points indicates the degree of similarity between communities.

Mantel tests showed that island and mainland sites were not spatially auto-correlated, as dissimilarities in liana community composition were not significantly related to geographic distances among island and mainland sites (r = 0.07, *p* = 0.18). Both abundance- and incidence-based NMDS ordinations indicated that there was no obvious pattern of convergence in community composition between larger islands (where area- and edge-effects are thought to be weakest) and mainland sites (Fig 5a and 5b). When environmental descriptors of island plots were considered, Area and Fire were the only significant predictors (*p* < 0.05) in abundance-based ordinations, and Area and Isolation were significant (*p* < 0.05) in incidence-based ordinations. However perMANOVA analyses, accounting for our nested sapling design, revealed that there was no significant difference between the community composition of island and mainland plots for both abundance- and incidence-data (S4 Table). Similarly, when exploring community composition among island plots, perMANOVA analyses did not reveal any significant environmental predictors for either abundance- or incidence-based compositions (S4 Table).

## Discussion

We investigated community-wide liana responses to landscape-scale forest fragmentation induced by a mega-dam, and our results demonstrate that liana communities remain remarkably intact despite insularization caused by reservoir filling. Our results also suggest that lianas are able to recruit more readily than trees in the most degraded habitats, given that we recorded more lianas than trees in the sapling layer on small, severely burned islands with little surrounding forest cover. Therefore as pervasive area- and edge-effects continue to act upon remnant forest isolates, future forest communities are likely to be increasingly dominated by lianas, with negative implications for tree community diversity and function. We propose that our findings provide further evidence of negative long-term biodiversity and carbon storage impacts of forest fragmentation induced by an Amazonian mega-dam.

### Increased dominance of young lianas in degraded forest sites

We found that recruitment of lianas into the sapling community appeared higher than recruitment of tree saplings in the most degraded insular forest habitats, and that lianas did not exhibit any evidence of community collapse across the Balbina archipelago. Liana compositional profiles remained remarkably consistent across both insular and continuous forest sites, in contrast to a rapidly eroding tree community [3]. Such a pattern suggests that lianas will likely become a dominant feature of reservoir land-bridge islands in the long term, and that liana communities appear to be robust to many of the negative impacts associated with landscape-scale habitat fragmentation [61,62]. A compositionally intact liana community could potentially exacerbate the erosion of remnant tree communities through direct and indirect competition [7,18,63]. Even in forest fragments embedded within a terrestrial habitat matrix, liana proliferation severely impacts tree communities and reduces carbon storage [64]. Given that reservoir land-bridge islands are the worst-case scenario in terms of fragmentation effects on remnant taxa [34], liana-induced reductions in tree diversity and carbon storage will likely be even more severe on islands than observed for forest fragments in a terrestrial landscape.

The dominance of liana saplings relative to tree saplings was elevated on small (< 10 ha) islands with low surrounding forest cover, particularly when fire disturbance was high, where lianas could apparently recruit more successfully than trees. Our findings are in line with previous studies where liana recruitment increased markedly in degraded forest fragments, edges and gaps, compared to tree recruitment [9,22,65]. However, the apparent increase in liana saplings relative to tree saplings appears to be the product of a decline in tree sapling recruitment, as liana sapling abundances remained largely consistent across island and mainland sites, indicating that tree saplings rely on higher quality forest in order to recruit successfully [65]. Nevertheless, successful recruitment of lianas in highly degraded islands will likely continue due to their competitive ability to establish in, for instance, the high light regimes associated with low-stature forests and higher density of canopy gaps as remnant forests continue to degrade [22,25,65]. Tree communities on small Balbina islands are structurally degraded and have reduced diversity [3]. Thus, liana recruitment will doubtless continue to increase relative to tree recruitment as island time increases, leading to highly liana-dominated forest communities in the future. Repeated surveys of the sapling layer would be needed to thoroughly investigate recruitment rates, as our study provides a snapshot of floristic communities ~30 years after island creation.

### Influence of forest cover and fire severity on mature lianas

In contrast to the sapling layer, we found that there were significantly fewer mature lianas on islands compared to the mainland, yet their total basal area did not significantly differ. The abundance of mature lianas also declined with fire severity, and increased with the amount of forest habitat surrounding islands. Such patterns indicate that low mature liana abundances on islands have been induced by a rapid turnover, and even loss, of canopy trees [3]. Lianas rely on large trees for structural support, and thus the abundance of mature lianas is lower in insular tree communities that have been most impacted by area- and edge-effects, and no-longer support tall closed-canopy forests [3,66]. As forest quality and vertical structure continues to decline across islands, we may therefore see concurrent degradation of the mature liana community in the long-term. However, lianas often circumvent the problem of loss of structural support through their ability to grow horizontally or along the ground [10]. Even high-climbing lianas can form low-lying tangles after their host trees fall, which continue to expand without the need for structural support from trees. Mature lianas will therefore likely persist across even the most degraded islands, further suppressing tree regeneration through competition [14,22].

The lower abundances of mature lianas on islands may also result from potential under-sampling of the mature liana community, as our sampling methodology only considered lianas growing vertically. On islands, where lianas lack sufficient trees for vertical growth, we may have under-estimated the abundance of mature lianas forced to grow horizontally or along the ground. This limitation also extends to under-sampling of non-vertical liana saplings. Yet even considering this potential under-sampling of liana saplings, we find strong evidence for increased recruitment of lianas into the sapling layer compared to trees on small and highly disturbed islands. We suggest that our evidence for heightened liana sapling recruitment and persistence of mature lianas, will continue to impact recruitment of both pioneer and shade-tolerant tree species on highly disturbed islands [18,67].

### Long-term persistence of lianas on semi-defaunated islands

The long-term persistence of lianas across this fragmented landscape may be further enhanced by the ability of vertebrate-dispersed lianas to persist, even in small semi-defaunated islands [38,68,43,69]. There was no significant difference in the proportion of biotically- vs. abiotically-dispersed stems found on islands compared to mainland sites, but most lianas surveyed were represented by two wind-dispersed genera. Liana communities may therefore shift towards being dominated by abiotically-dispersed genera over time, but the ability for biotically-dispersed lianas to reproduce vegetatively may temper this shift [70]. In contrast, studies have demonstrated that biotically-dispersed trees show significant declines through habitat fragmentation and loss of vertebrate dispersers [41], and in Balbina, biotically-dispersed trees have been strongly affected by fire disturbance across the archipelago [3]. The persistence of liana assemblages in the aftermath of landscape-scale fragmentation, and loss of vertebrate dispersers, is echoed by similarities in liana diversity between island and mainland sites. Although liana diversity declined in increasingly isolated islands, the most distant islands exhibited patterns of diversity within the range of mainland sites.

### Similarity of liana community composition among islands and mainland continuous forest

Despite diverse disturbance regimes, island and mainland plots have remarkably consistent liana community compositions. Again, this is in stark contrast to, for example, the adult tree community within Balbina, which has undergone drastic compositional shifts due to area- and edge-effects [3]. The absence of any clear patterns of liana community composition related to area, isolation and fire disturbance could be due to low taxonomic resolution, as we only identified lianas to the level of genus [71,72]. However when genus-level information for tree saplings was used in comparable analyses, we found that tree saplings displayed strikingly different responses, such as significant increases in diversity with island area, and clear separation of community composition between the most highly disturbed islands and mainland continuous forest sites, following similar patterns seen at species-level [3]. We therefore conclude that the observed similarities of liana community compositions are ‘true’ patterns, rather than artefacts of low taxonomic resolution.

### Drivers of liana assemblage responses to insularization

Given that the species richness and functional diversity of adult tree assemblages in Balbina significantly increased with island area [3], we expected that area would be similarly influential in predicting the community attributes and composition of lianas across this fragmented landscape, through species-area effects [73]. Instead, the amount of forest habitat surrounding isolates, and historical fire disturbance, acted synergistically as the strongest drivers determining the abundance and dominance of lianas relative to trees [74–76]. In studies of liana communities in forest fragments embedded within a terrestrial matrix, historical forest disturbance was also an important driver of liana abundance and diversity, as was distance to forest edges and soil properties [9,77].

## Conclusions

We show that liana communities persist across a man-made archipelagic landscape, and increase in abundance and dominance relative to trees in both sapling and mature size classes. Diversity and community composition of lianas were apparently relatively unaffected by insularization, and there were no compositional differences between island and mainland forest sites. We stress that given the continuing decline of tree communities on hydroelectric reservoir land-bridge islands [3,34], a persistent liana community will likely exacerbate the degradation of remnant tree communities through direct and indirect competition in the long term [6,18,78]. Declines in remnant tree communities not only impact faunal populations [43], but also the carbon cost/benefit calculations of dams [79]. Dams in lowland tropical forest regions are controversial in terms of their net carbon budget, in which losses are not always offset by their ‘green energy’ production [80,81]. Reservoir islands are not currently accounted for in environmental impact assessments of new dam proposals [82,83], and declines in both the biodiversity value and carbon storage capacity of reservoir islands could be exacerbated by elevated liana abundance and dominance relative to trees [20].

## Supporting information

S1 Table Overview of lianas and trees inventoried. Number of sapling and mature lianas, number of liana genera, and number of tree saplings and adults within all 89 plots inventoried across 36 islands and three mainland continuous forest sites across the Balbina Hydroelectric Dam landscape (Brazilian Amazon). NS = not surveyed.
(DOCX)

S2 Table Overview of liana seed dispersal modes. Liana sapling genera with total abundances and seed dispersal mode classification.
(DOCX)

S3 Table Summary of ANOVA results. ANOVA testing overall differences between island and mainland plots.
(DOCX)

S4 Table Overview of perMANOVA analysis. perMANOVA tests of abundance- and incidence-based community compositions, which were carried out between island and mainland plots, and among island plots with environmental variables. perMANOVA for abundance-based compositional data was carried out using dissimilarities derived from the Morisita-Horn dissimilarity index, and for incidence-based composition using Jaccard dissimilarities.
(DOCX)
