# Supplementary material for: Woody lianas increase in dominance and maintain compositional integrity across an Amazonian dam-induced fragmented landscape
Source: PLoS One. 2017 Oct 17;12(10):e0185527. doi: 10.1371/journal.pone.0185527 (PMC5644977; doi:10.1371/journal.pone.0185527)
Supplement: S1 Table — Number of sapling and mature lianas, number of liana genera, and number of tree saplings and adults within all 89 plots inventoried across 36 islands and three mainland continuous forest sites across the Balbina Hydroelectric Dam landscape (Brazilian Amazon). NS = not surveyed. (DOCX) [file pone.0185527.s001.docx]

**S1 Table. Overview of lianas and trees inventoried.** Number of sapling and mature lianas, number of liana genera, and number of tree saplings and adults within all 89 plots inventoried across 36 islands and three mainland continuous forest sites across the Balbina Hydroelectric Dam landscape (Brazilian Amazon). NS = not surveyed.

| **Island** | **Island location (UTM)** | | **Area (ha)** | **Plot number** | **Number of liana saplings** | **Number of liana sapling genera** | **Number of mature lianas** | **Number of tree saplings** | **Number of adult trees** |
| --- | --- | --- | --- | --- | --- | --- | --- | --- | --- |
|  | **Long.** | **Lat.** |  |  |  |  |  |  |  |
| Abandonada | 190832 | 9845594 | 0.65 | 1 | 14 | 8 | NS | 27 | 14 |
| Toquinho | 193044 | 9809871 | 0.83 | 1 | 29 | 5 | 5 | 30 | 14 |
| Joaninha | 185199 | 9831509 | 1.15 | 1 | 16 | 7 | 0 | 41 | 21 |
| Xibé | 184382 | 9837441 | 1.45 | 1 | 27 | 13 | 8 | 130 | 57 |
| Formiga | 230687 | 9797172 | 1.52 | 1 | 9 | 3 | 14 | 16 | 10 |
| Andre | 180404 | 9824636 | 2.17 | 1 | 12 | 6 | 11 | 67 | 38 |
| Lozivaldo | 228480 | 9795658 | 2.39 | 1 | 70 | 17 | NS | 41 | 10 |
| Cafundó | 209556 | 9833953 | 2.7 | 1 | 10 | 6 | 23 | 44 | 24 |
| Panema | 200445 | 9803641 | 3.53 | 1 | 30 | 10 | 12 | 110 | 40 |
| Torem | 207076 | 9797590 | 3.94 | 1 | 26 | 10 | 0 | 43 | 27 |
| Pé Torto | 237085 | 9804582 | 5.85 | 1 | 50 | 11 | 15 | 74 | 36 |
| Jiquitaia | 211241 | 9796880 | 7.28 | 1 | 23 | 8 | 27 | 92 | 50 |
| Arrepiado | 195202 | 9832395 | 8.35 | 1 | 30 | 8 | 28 | 95 | 43 |
| Garrafa | 184561 | 9824221 | 9.54 | 1 | 28 | 5 | 17 | 96 | 48 |
| Abusado | 201965 | 9805012 | 13.41 | 1 | 24 | 10 | 30 | 116 | 57 |
| Abusado | 201965 | 9805012 | 13.41 | 2 | 34 | 12 | 19 | 105 | 50 |
| Piquiá | 189705 | 9833233 | 13.59 | 1 | 64 | 15 | 16 | 133 | 39 |
| Piquiá | 189705 | 9833233 | 13.59 | 2 | 76 | 14 | 21 | 138 | 57 |
| Coatá | 189850 | 9835180 | 17.45 | 1 | 25 | 10 | 28 | 138 | 62 |
| Coatá | 189850 | 9835180 | 17.45 | 2 | 36 | 9 | 53 | 128 | 67 |
| Palhal | 227654 | 9802034 | 21.21 | 1 | 15 | 6 | 14 | 44 | 27 |
| Palhal | 227654 | 9802034 | 21.21 | 2 | 11 | 7 | 19 | 53 | 23 |
| Neto | 238178 | 9796466 | 32.92 | 1 | 49 | 12 | 26 | 184 | 71 |
| Neto | 238178 | 9796466 | 32.92 | 2 | 31 | 10 | 45 | 125 | 61 |
| Bacaba | 185861 | 9833944 | 53.3 | 1 | 25 | 10 | 12 | 99 | 48 |
| Bacaba | 185861 | 9833944 | 53.3 | 2 | 19 | 10 | 24 | 115 | 43 |
| Relógio | 205233 | 9814450 | 72.1 | 1 | 45 | 7 | 37 | 71 | 32 |
| Relógio | 205233 | 9814450 | 72.1 | 2 | 21 | 8 | 19 | 76 | 39 |
| Sapupara | 208845 | 9812245 | 78.44 | 1 | 15 | 7 | 18 | 117 | 54 |
| Sapupara | 208845 | 9812245 | 78.44 | 2 | 39 | 12 | 35 | 143 | 71 |
| Adeus | 204386 | 9792495 | 97.62 | 1 | 51 | 9 | 15 | 83 | 44 |
| Adeus | 204386 | 9792495 | 97.62 | 2 | 54 | 14 | 24 | 136 | 72 |
| Moita | 178208 | 9827785 | 98.84 | 1 | 18 | 9 | 20 | 87 | 50 |
| Moita | 178208 | 9827785 | 98.84 | 2 | 23 | 8 | 17 | 154 | 64 |
| Moita | 178208 | 9827785 | 98.84 | 3 | 15 | 4 | 15 | 166 | 61 |
| Pontal | 200900 | 9797360 | 110.43 | 1 | 41 | 12 | 38 | 158 | 81 |
| Pontal | 200900 | 9797360 | 110.43 | 2 | 34 | 11 | 18 | 163 | 66 |
| Pontal | 200900 | 9797360 | 110.43 | 3 | 18 | 7 | 27 | 156 | 70 |
| Furo de Santa Luzia | 228288 | 9807602 | 193 | 1 | 36 | 9 | 18 | 110 | 62 |
| Furo de Santa Luzia | 228288 | 9807602 | 193 | 2 | 39 | 9 | 35 | 171 | 68 |
| Furo de Santa Luzia | 228288 | 9807602 | 193 | 3 | 35 | 10 | 24 | 149 | 65 |
| Cipoal | 190016 | 9811616 | 218.74 | 1 | 56 | 9 | 32 | 143 | 61 |
| Cipoal | 190016 | 9811616 | 218.74 | 2 | 68 | 14 | 15 | 136 | 67 |
| Cipoal | 190016 | 9811616 | 218.74 | 3 | 56 | 9 | 19 | 162 | 78 |
| Jabuti | 193340 | 9819811 | 231.39 | 1 | 21 | 6 | 22 | 128 | 53 |
| Jabuti | 193340 | 9819811 | 231.39 | 2 | 21 | 9 | 22 | 137 | 61 |
| Jabuti | 193340 | 9819811 | 231.39 | 3 | 21 | 7 | 24 | 192 | 76 |
| Tucumari | 229191 | 9823073 | 292.41 | 1 | 9 | 4 | 37 | 82 | 52 |
| Tucumari | 229191 | 9823073 | 292.41 | 2 | 20 | 7 | 37 | 84 | 53 |
| Tucumari | 229191 | 9823073 | 292.41 | 3 | 27 | 13 | 25 | 105 | 46 |
| Martelo | 197665 | 9815138 | 471 | 1 | 30 | 9 | 29 | 155 | 64 |
| Martelo | 197665 | 9815138 | 471 | 2 | 16 | 5 | 27 | 112 | 65 |
| Martelo | 197665 | 9815138 | 471 | 3 | 21 | 10 | 43 | 108 | 45 |
| Tristeza | 193659 | 9805664 | 487.5 | 1 | 42 | 10 | 10 | 96 | 41 |
| Tristeza | 193659 | 9805664 | 487.5 | 2 | 64 | 14 | 21 | 125 | 65 |
| Tristeza | 193659 | 9805664 | 487.5 | 3 | 49 | 15 | 47 | 216 | 74 |
| Tristeza | 193659 | 9805664 | 487.5 | 4 | 45 | 11 | 66 | 161 | 63 |
| Beco Catitu | 199083 | 9807374 | 637.49 | 1 | 31 | 12 | 29 | 158 | 65 |
| Beco Catitu | 199083 | 9807374 | 637.49 | 2 | 44 | 16 | 15 | 175 | 54 |
| Beco Catitu | 199083 | 9807374 | 637.49 | 3 | 22 | 8 | 15 | 92 | 54 |
| Beco Catitu | 199083 | 9807374 | 637.49 | 4 | 29 | 10 | 14 | 192 | 74 |
| Mascote | 184723 | 9817351 | 673.35 | 1 | 23 | 9 | 14 | 122 | 67 |
| Mascote | 184723 | 9817351 | 673.35 | 2 | 10 | 5 | 22 | 95 | 50 |
| Mascote | 184723 | 9817351 | 673.35 | 3 | 28 | 10 | 31 | 240 | 67 |
| Mascote | 184723 | 9817351 | 673.35 | 4 | 13 | 7 | 17 | 165 | 66 |
| Fuzaca | 181196 | 9834327 | 761.02 | 1 | 37 | 15 | 46 | 151 | 78 |
| Fuzaca | 181196 | 9834327 | 761.02 | 2 | 35 | 11 | 19 | 92 | 59 |
| Fuzaca | 181196 | 9834327 | 761.02 | 3 | 23 | 14 | 41 | 111 | 65 |
| Fuzaca | 181196 | 9834327 | 761.02 | 4 | 37 | 11 | 14 | 139 | 68 |
| Porto Seguro | 220105 | 9803627 | 1466 | 1 | 42 | 14 | 33 | 146 | 65 |
| Porto Seguro | 220105 | 9803627 | 1466 | 2 | 30 | 7 | 38 | 131 | 59 |
| Porto Seguro | 220105 | 9803627 | 1466 | 3 | 27 | 10 | 40 | 114 | 51 |
| Porto Seguro | 220105 | 9803627 | 1466 | 4 | 35 | 10 | 34 | 131 | 63 |
| Gavião-Real | 207890 | 9824114 | 1690.04 | 1 | 23 | 7 | 19 | 119 | 49 |
| Gavião-Real | 207890 | 9824114 | 1690.04 | 2 | 44 | 11 | 31 | 138 | 44 |
| Gavião-Real | 207890 | 9824114 | 1690.04 | 3 | 23 | 7 | 20 | 115 | 55 |
| Gavião-Real | 207890 | 9824114 | 1690.04 | 4 | 27 | 11 | 35 | 85 | 52 |
| Mainland 1 | 196178 | 9795056 | NA | 1 | 39 | 9 | 21 | 185 | 65 |
| Mainland 1 | 196178 | 9795056 | NA | 2 | 37 | 11 | 42 | 147 | 67 |
| Mainland 1 | 196178 | 9795056 | NA | 3 | 34 | 9 | 51 | 137 | 66 |
| Mainland 1 | 196178 | 9795056 | NA | 4 | 20 | 9 | 31 | 147 | 75 |
| Mainland 2 | 250891 | 9798804 | NA | 1 | 30 | 8 | 40 | 149 | 82 |
| Mainland 2 | 250891 | 9798804 | NA | 2 | 20 | 10 | 32 | 168 | 45 |
| Mainland 2 | 250891 | 9798804 | NA | 3 | 78 | 13 | 27 | 240 | 89 |
| Mainland 2 | 250891 | 9798804 | NA | 4 | 41 | 11 | 69 | 189 | 64 |
| Mainland 3 | 177529 | 9845108 | NA | 1 | 29 | 13 | 32 | 130 | 66 |
| Mainland 3 | 177529 | 9845108 | NA | 2 | 38 | 13 | 30 | 118 | 64 |
| Mainland 3 | 177529 | 9845108 | NA | 3 | 64 | 19 | 39 | 179 | 84 |
| Mainland 3 | 177529 | 9845108 | NA | 4 | 20 | 9 | 16 | 181 | 76 |
