# Supplementary material for: Woody lianas increase in dominance and maintain compositional integrity across an Amazonian dam-induced fragmented landscape
Source: PLoS One. 2017 Oct 17;12(10):e0185527. doi: 10.1371/journal.pone.0185527 (PMC5644977; doi:10.1371/journal.pone.0185527)
Supplement: S2 Table — Liana sapling genera with total abundances and seed dispersal mode classification. (DOCX) [file pone.0185527.s002.docx]

**S2 Table. Overview of liana seed dispersal modes.** Liana sapling genera with total abundances and seed dispersal mode classification.

| **Genus** | **Seed dispersal mode** | **Number of liana saplings** |
| --- | --- | --- |
| *Abuta* | Biotically | 73 |
| *Ampelozizyphus* | Abiotically | 17 |
| *Bauhinia* | Abiotically | 107 |
| *Cheiloclinio* | Biotically | 84 |
| *Cissus* | Biotically | 1 |
| *Cocoloba* | Biotically | 1 |
| *Connarus* | Abiotically | 1 |
| *Cupania* | Biotically | 11 |
| *Davila* | Biotically | 126 |
| *Derris* | Abiotically | 215 |
| *Dioclea* | Abiotically | 61 |
| *Doliocarpus* | Biotically | 73 |
| *Ficus* | Biotically | 1 |
| *Gurania* | Biotically | 6 |
| *Macherium* | Abiotically | 553 |
| *Mansoa* | Abiotically | 207 |
| *Memora* | Abiotically | 552 |
| *Mimosa* | Abiotically | 8 |
| *Moutabea* | Biotically | 315 |
| *Mucuna* | Abiotically | 62 |
| *Odontadenia* | Abiotically | 23 |
| *Passiflora* | Biotically | 34 |
| *Paulinia* | Biotically | 9 |
| *Salacia* | Biotically | 242 |
| *Senna* | Abiotically | 27 |
| *Serjania* | Abiotically | 9 |
| *Smilax* | Biotically | 12 |
| *Stigmaphyllon* | Abiotically | 4 |
| *Strychnos* | Biotically | 27 |
| *Uncarium* | Abiotically | 4 |
| *Vitis* | Biotically | 1 |
