# Supplementary material for: Woody lianas increase in dominance and maintain compositional integrity across an Amazonian dam-induced fragmented landscape
Source: PLoS One. 2017 Oct 17;12(10):e0185527. doi: 10.1371/journal.pone.0185527 (PMC5644977; doi:10.1371/journal.pone.0185527)
Supplement: S3 Table — ANOVA testing overall differences between island and mainland plots. (DOCX) [file pone.0185527.s003.docx]

**S3 Table.** **Summary of ANOVA results.** ANOVA testing overall differences between island and mainland plots.

|  | **SS** | **df** | **Mean Square** | **F** | ***P*** |
| --- | --- | --- | --- | --- | --- |
| Abundance of sapling lianas | 389.3 | 1 | 389.28 | 1.59 | 0.21 |
| Abundance of mature lianas | 1352 | 1 | 1352.3 | 8.97 | 0.004 |
| Total basal area of mature lianas | 0.006 | 1 | 0.006 | 0.57 | 0.45 |
| Ratio biotic : abiotically dispersed stems | 0.65 | 1 | 0.65 | 3.58 | 0.06 |
| Relative dominance of liana saplings : tree saplings | 0.06 | 1 | 0.064 | 1.43 | 0.23 |
| Relative dominance of mature lianas : trees >10cm DBH | 0.05 | 1 | 0.05 | 4.66 | 0.03 |
| Fisher’s α | 4.85 | 1 | 4.85 | 1.02 | 0.31 |
