# Supplementary material for: Woody lianas increase in dominance and maintain compositional integrity across an Amazonian dam-induced fragmented landscape
Source: PLoS One. 2017 Oct 17;12(10):e0185527. doi: 10.1371/journal.pone.0185527 (PMC5644977; doi:10.1371/journal.pone.0185527)
Supplement: S4 Table — perMANOVA tests of abundance- and incidence-based community compositions, which were carried out between island and mainland plots, and among island plots with environmental variables. perMANOVA for abundance-based compositional data was carried out using dissimilarities derived from the Morisita-Horn dissimilarity index, and for incidence-based composition using Jaccard dissimilarities. (DOCX) [file pone.0185527.s004.docx]

**S4 Table.** **Overview of perMANOVA analysis.** perMANOVA tests of abundance- and incidence-based community compositions, which were carried out between island and mainland plots, and among island plots with environmental variables. perMANOVA for abundance-based compositional data was carried out using dissimilarities derived from the Morisita-Horn dissimilarity index, and for incidence-based composition using Jaccard dissimilarities. To account for a nested sampling design, ‘site’ was set as a grouping variable within the perMANOVA.

|  |  | **Df** | **SS** | **F** | **R^2^** | ***P*** |
| --- | --- | --- | --- | --- | --- | --- |
| Island vs. mainland | Abundance  Incidence | 1  1 | 0.29  0.4 | 3.35  1.68 | 0.04  0.02 | >0.05  >0.05 |
| Area | Abundance  Incidence | 1  1 | 0.19  0.44 | 2.21  1.84 | 0.02  0.02 | >0.05  >0.05 |
| Isolation | Abundance  Incidence | 1  1 | 0.41  0.64 | 4.91  2.68 | 0.06  0.03 | >0.05  >0.05 |
| Cover | Abundance  Incidence | 1  1 | 0.08  0.28 | 0.95  1.19 | 0.011  0.015 | >0.05  >0.05 |
| Fire | Abundance  Incidence | 2  2 | 0.28  0.66 | 0.82  0.32 | 0.02  1.37 | >0.05  >0.05 |
